# Anti-Menopausal Effect of Soybean Germ Extract and *Lactobacillus gasseri* in the Ovariectomized Rat Model

**DOI:** 10.3390/nu15204485

**Published:** 2023-10-23

**Authors:** Sun-Hee Lee, Tae-Joong Lim, Eun Ju Yun, Kyoung Heon Kim, Sanghyun Lim

**Affiliations:** 1 Department of Biotechnology, Graduate School, Korea University, Seoul 02841, Republic of Korea; wowshl@korea.ac.kr; 2 R&D Center, Cell Biotech Co., Ltd., Gimpo 10003, Republic of Korea; dirwillow@naver.com; 3 Division of Biotechnology, Jeonbuk National University, Iksan 54596, Republic of Korea; ejyun@jbnu.ac.kr

**Keywords:** menopause, soybean germ extract, isoflavone, probiotics, lactic acid bacteria, aglycone

## Abstract

Menopause is a significant phase in a woman’s life. Menopausal symptoms can affect overall well-being and quality of life. Conventionally, hormone replacement therapy (HRT) is used to alleviate menopausal symptoms; however, depending on the conditions, HRT may lead to side effects, necessitating the exploration of alternative therapies with fewer side effects. In this study, we investigated the effects of a combination of soybean germ extract (S30) containing 30% (*w*/*w*) isoflavone and a probiotic, *Lactobacillus gasseri* (LGA1), on menopausal conditions in an ovariectomized (OVX) rat model. We evaluated the impact of S30+LGA on body weight, estrogen markers, uterine and bone health, vascular markers, and neurotransmitter levels. The results revealed that treatment with S30+LGA1 significantly improved body weight and uterine and bone health. Moreover, S30+LGA1 demonstrated promising effects on lipid profile, liver function, and vascular markers and positively impacted serotonin and norepinephrine levels, indicating potential mood-enhancing effects. In conclusion, S30+LGA1, possessing anti-menopausal effects in vitro and in vivo, can be recommended as a soy-based diet, which offers various health benefits, especially for menopausal women.

## 1. Introduction

Menopause results from a gradual decline in estrogen levels. It is associated with an increased risk of experiencing emotional disorders, such as anxiety and depression [1]. Moreover, post-menopausal reduction in estrogen levels is linked to an increased likelihood of developing conditions such as coronary artery and cardiovascular diseases, osteoporosis, urinary tract infections and incontinence, weight gain, and reduced neuroprotective effects [2,3,4,5].

Hormone replacement therapy (HRT), or estrogen and progesterone supplementation, is commonly used to alleviate menopausal symptoms [6,7]. The advantages and disadvantages of HRT vary depending on factors such as age, menopause symptoms, and specific risk factors [8,9]. Therefore, the development of alternative therapies, which can provide health benefits to menopausal women without causing side effects, remains a pressing priority.

Soybeans are common in Asian diets and are rich in phytoestrogens. A correlation between soybean consumption and reduced occurrence of menopausal symptoms, osteoporosis, cardiovascular diseases, and hormone-dependent cancers has been demonstrated [10]. These benefits can be attributed to the presence of isoflavones—a type of phytoestrogen [11]. Isoflavones are structurally similar to 17β-estradiol [12] and mimic the action of estrogen on organs by binding to estrogen receptors [13]. Soy isoflavones exist primarily as β-glycosides and their acetyl and malonyl conjugates [14]. However, β-glycosides have a high polarity and poor bioavailability, and a saccharide group in their structure limits their absorption [15]. Therefore, β-glycosides need to be hydrolyzed into aglycones with higher bioavailability than isoflavone, such as daidzein and genistein [2,16,17], catalyzed by intestinal or bacterial enzymes [18,19,20]. The impact of isoflavones on human health is considerably influenced by the composition of the gut microbiota in the host [11,21,22,23]. Therefore, gut microbiota must be utilized to convert soy isoflavones into aglycones for enhanced health benefits [24,25].

Gut microbiota actively participate in food metabolism via the expression of various enzymes [26]. Gut microbiota contribute to the metabolism of isoflavones [27]. Intestinal microbiota cleave the glycosidic bond in glycosides, resulting in their conversion to aglycones [2,21,28,29,30,31]. This enzymatic action is primarily performed by bacterial β-glycosidases [2,21,28,29,30,31]. Lactic acid bacteria (LAB) are capable of producing β-glucosidases [32,33]. Therefore, LAB can be important tools for enhancing the health benefits of isoflavones.

This study primarily aimed to identify, via screening, LAB with high β-glucosidase activity, which can convert soy isoflavone glucosides into their active aglycone forms to enhance their bioavailability and health benefits. In addition, the potential therapeutic effects of soybean germ extract and *Lactobacillus gasseri* were investigated using in vitro and in vivo models of menopause.

## 2. Materials and Methods

### 2.1. Preparation of S30

The S30 was prepared by dissolving 40 g of soybean germ extract (isoflavone 30%, S30, Seorim Bio Inc., Busan, Korea) supplied by Mirae Biotech Co. (Pocheon, Korea), in sterilized water. The pH of the S30 was adjusted to 6.7 using 5 M sodium hydroxide, and it was then sterilized via autoclaving at 121 °C for 15 min.

### 2.2. Preparation of Bacterial Species Culture

*Lactobacillus rhamnosus* CBT LR5 (KCTC 12202BP, LR5), *L. gasseri* CBT LGA1 (KCTC 12936BP, LGA1), and *Lactococcus lactis* subsp. *Lactis* CBT SL6 (KCTC 11865BP, SL6) were cultured in De Man, Rogosa, and Sharpe medium (Difco, Detroit, MI, USA) at 37 °C for 18 h aerobically. *Bifidobacterium breve* CBT BR3 (KCTC 12201BP, BR3) and *B. longum* CBT BG7 (KCTC 12200BP, BG7) were cultured in BL^®^ medium (Difco, Detroit, MI, USA) at 37 °C for 18 h anaerobically. After incubation, the probiotic cultures were centrifuged, and the resulting pellets containing 1 × 10^9^ CFU/mL were obtained. Then, the pellets were washed thrice using a 100 mM potassium phosphate buffer (pH 7.4) and used for the β-glucosidase enzyme activity test.

### 2.3. Assay for β-Glucosidase Enzyme from Probiotics in S30

After the final wash, the bacterial cells were resuspended in 1 mL of sterilized water, and the optical density (OD) at 600 nm was adjusted to the same level for each probiotic species. Subsequently, the pellets were inoculated into 20 mL of S30 solution. The pellets were collected at specific time points: 1, 2, 4, 8, 12, 24, and 48 h. The ODs of all species were adjusted to match the same lowest OD value. Subsequent time points were measured based on the OD value in the first hour, and OD values were standardized for each species. The enzyme activity in S30 was determined by measuring the hydrolysis rate of *p*-nitrophenyl-β-d-glucopyranoside (*p*NPG). The pellets were resuspended with 1 mL of a 5 mM *p*NPG solution in 100 mM sodium phosphate buffer (pH 7.0) and incubated at 37 °C for 15 min [34]. The reaction was stopped by adding 0.5 mL of 1 M cold sodium carbonate. Subsequently, the reaction mixture was transferred to 1.8 mL Eppendorf tubes and centrifuged at 14,000× *g* for 30 min. Finally, the amount of *p*-nitrophenol released from *p*NPG was measured at 420 nm using a spectrophotometer (Pharmacia LKB, Novospec II, Uppsala, Sweden). One unit of β-glucosidase activity was defined as the amount of enzyme required to produce 1 μmole of *p*-nitrophenol from the *p*NPG per minute under the reaction conditions described above.

### 2.4. Cell Culture

MCF-7, the human breast adenocarcinoma cell line, was purchased from the American Tissue Culture Collection (ATCC, Manassas, VA, USA) and cultured in Dulbecco’s Modified Essential Medium supplemented with 10% fetal bovine serum (FBS), 100 IU/mL of penicillin G, and 100 µg/mL of streptomycin. The human osteosarcoma cell line, MG-63, was obtained from ATCC and cultured in a minimal essential medium supplemented with 10% FBS and the same concentrations of penicillin G and streptomycin. Cultures in exponential growth were maintained at 37 °C in a humidified atmosphere with 5% CO_2_.

### 2.5. Real-Time Reverse-Transcription PCR Assay

After a 24 h treatment with fermentation products obtained from filtered probiotics, the MCF-7 and MG-63 cells were immediately collected, frozen in liquid nitrogen (LN), and stored at −80 °C. Then, these frozen cells were homogenized using an LN-cooled mortar, and RNA was extracted using Trizol (Invitrogen, Vacaville, CA, USA) according to the manufacturer’s instructions. The obtained RNA underwent reverse transcription using HyperScript reverse transcription reagents (GeneAll Biotechnology, Seoul, Korea). After adding SYBR Green Supermix (iQ SYBR Green Supermix, Bio-Rad, Hercules, CA, USA) to the reaction mixture, real-time quantitative PCR (qPCR) was performed using a Roche LightCycler^®^ 480 Real-Time PCR System (Roche, Basel, Switzerland). The primers used in this study are listed in Table S1. The amplification process involved an initial hold at 95 °C for 10 min, followed by 40 cycles of 30 s at 95 °C and 1 min at 60 °C. The samples were subjected to triple amplification on the same plate, along with appropriate controls. Detection of primer dimers and other artifacts was achieved through analysis of the dissociation curve. The 2^−ΔΔCt^ method was used to calculate the relative expression of target genes, using β-actin as the endogenous gene for normalization.

### 2.6. Animal Models

Thirty-six female Sprague Dawley rats, aged 6 weeks, were obtained from Orient Bio (Seoul, Korea). The rats were maintained in a separate cage in a controlled environment with a temperature of 24 ± 2 °C, relative humidity of 40–60%, and a 12 h light/dark cycle.

The rats were provided ad libitum access to food and water during the 1-week adaptation period. Subsequently, the rats underwent bilateral ovariectomies (OVX) under anesthesia (pentobarbital sodium). Sham-operated rats (sham) underwent the same surgical procedure, except without ovariectomy. One week post-operatively, the rats were randomly divided into four groups based on their weight, with 10 animals in each group. The rats were fed a modified version of the AIN-93G diet, where corn oil was replaced with soybean oil.

**Figure 1:**
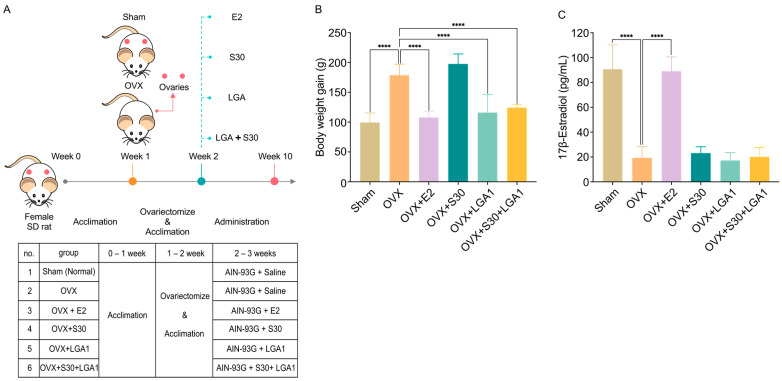
Phenotypes of OVX rats. (**A**) Schematics of experimental design. (**B**) Effect of S30+LGA1 on body weight in OVX rats. (**C**) Effect of S30+LGA1 on 17β-Estradiol in OVX rats. Data are presented as the mean ± standard deviation (SD). **** *p* < 0.0001 compared with the OVX group. OVX, ovariectomized; E2, 17β-Estradiol; S30, soybean germ extract; LGA1, *Lactobacillus gasseri*; SD rat, Sprague Dawley rat.

The rats were further assigned randomly to one of six groups, with six rats in each group (Figure 1A): (1) sham group, administered PBS; (2) OVX group, administered PBS; (3) OVX + 17β-estradiol (E2) group, administered intraperitoneal injections of 10 μg/kg of 17β-estradiol thrice a week; (4) OVX + S30 group, orally administered S30 at a dose of 10 mg/kg; (5) OVX + LGA1 group, administered LGA1 at a dose of 1 × 10^9^ CFU/head; and (6) OVX + S30 + LGA1 group, administered both S30 at a dose of 10 mg/kg and LGA1 at a dose of 1 × 10^9^ CFU/head. LGA1 was in a lyophilized powder form. S30 and LGA1 administration was continued for 8 weeks.

The animal study protocol was reviewed and approved by the Institutional Animal Care and Use Committee board (IACUC) at CellBiotech (Approval No: CBT-2020-01), following the guidelines of the Association for Assessment and Accreditation of Laboratory Animal Care (AAALAC).

### 2.7. Micro-Computerized Tomography Analysis

Micro-computerized tomography (micro-CT) images of the fixed left femur samples and a standard phantom were acquired using a micro-CT scanner (Skyscan 1176; BRUKER, Kontich, Belgium). The scanning parameters were set as follows: X-ray voltage, 50 kV; current, 445 μA; resolution, 18 μm; camera pixel size, 18.02 mm; aluminum filter size, 1 mm; thick aluminum filter was used; scanning process rotation step, 0.5°; and rotation angle, 360°. The acquired images were reconstructed using the image analyzer (Zen 3.1, Carl Zeiss, Jena, Germany;). Subsequently, the reconstructed images were analyzed using the same software. Various bone parameters of the left femur head, such as bone volume/total volume (BV/TV), bone mineral density (BMD), trabecular number (Tb. N), trabecular thickness (Tb. Th), and trabecular separation (Tb. Sp), were analyzed.

### 2.8. Measurement of Abdominal Fat in Live Animals

Abdominal visceral adiposity parameters of the live animals, including total abdominal fat (TAF), visceral abdominal fat (VAF), and subcutaneous abdominal fat (SAF) volume, were measured non-invasively at 12 weeks using three-dimensional (3D) Scanco VivaCT 80 scanner (Scanco Medical AG, Bassersdorf, Switzerland), following the manufacturer’s instructions.

During the scanning process, the rats were anesthetized with 1% isoflurane (inhalation) and positioned on their back with their face up and head toward the front. Both hind limbs were extended and secured to a specimen holder, forming a 90° angle between the femur and spine, with the legs fully extended. The scanning area covered the region between the proximal end of the L1 and the distal end of the L6 lumbar vertebrae. Scanning was performed with an isotropic voxel size of 18 μm, using parameters of 70 kVp energy, 114 μA intensity, 31.9 mm field of view (FOV)/diameter, and 200 ms integration time. The scanning energy and voxel size were determined based on optimizing scanning time, tissue detail, and minimizing radiation exposure, following the manufacturer’s guidelines.

The acquired micro-CT scans were processed using the imaging software (Scanco Medical AG, vivaCT 80, Bassersdorf, Switzerland), and the TAF, VAF, and SAF were analyzed using the generated 3D reconstructed images.

### 2.9. Analysis of the Uterine Morphometric Parameters

Sections of the uterus were stained with hematoxylin and eosin and prepared for light microscopy analysis. The radius of the uterine endometrium (including the luminal and glandular epithelia and lamina propria) and myometrium was determined via optical micrometry using a ×10 objective. Additionally, the height of the luminal epithelium was measured using a ×20 objective. Measurements were calibrated using the image of a stage micrometer at the same magnification to ensure standardization. Four areas within each of the three transverse sections of the uterus were analyzed for each animal. Regarding epithelial cell height, four measurements were taken within the aforementioned areas.

### 2.10. Serum Biochemical Marker Analysis

Serum levels of estradiol, serotonin, norepinephrine, osteocalcin (OC), bone alkaline phosphatase (ALP), deoxypyridinoline (DPD), pyridinoline (PYD), *C*-telopeptide of type I collagen (CTX-1), *N*-telopeptide of type I collagen (NTX-1), endothelin-1, and endothelial nitric oxide (eNOS) were analyzed using the corresponding specific ELISA kits for rats: rat Estradiol (E2) ELISA Kit (Cat. No. MBS702969; MyBioSource, San Diego, CA, USA), rat 5-hydroxy tryptamine (5-HT) ELISA Kit (Cat. No. MBS266539; MyBioSource), rat norepinephrine (NE) ELISA Kit (Cat. No. MBS269993; MyBioSource), rat osteocalcin (OC) ELISA Kit (Cat. No. MBS2022619; MyBioSource), rat bone alkaline phosphatase ELISA kit (Cat. No. MBS700904; MyBioSource), rat deoxypyridinoline (DPD) ELISA Kit (Cat. No. MBS702106; MyBioSource), rat pyridinoline (PYD) ELISA Kit (Cat. No. MBS2533532; MyBioSource), rat cross-linked *C*-telopeptide of type I collagen (CTX-I) ELISA Kit (Cat. No. MBS2020126; MyBioSource), rat cross-linked *N*-telopeptide of type I collagen, NTX-1 ELISA Kit (Cat. No. MBS703585; MyBioSource), rat endothelin 1 ELISA Kit (Cat. No. MBS733833; MyBioSource), and rat endothelial nitric oxide synthase ELISA Kit (Cat. No. MBS261741; MyBioSource). The serum samples were processed following the manufacturer’s instructions. The absorbance of samples obtained from each assay was measured using the VersaMaxTM tunable microplate reader (Molecular Device, San Jose, CA, USA).

Biochemical parameters, including cholesterol, triglycerides, high-density lipoprotein (HDL), low-density lipoprotein (LDL), aspartate aminotransferase (AST), alanine transaminase (ALT), albumin, and calcium levels, were measured using the XL-100 automatic analyzer (Erba, Germany).

### 2.11. Statistical Analysis

The data were presented as mean ± standard error of the mean (SEM). Statistical analysis was conducted using GraphPad Prism 9 software (GraphPad Software, La Jolla, CA, USA). The significance of differences was evaluated using a one-way analysis of variance (ANOVA) with Sidak multiple comparisons tests among multiple groups. In the figures, asterisks denote statistical significance (*, *p* < 0.05; **, *p* < 0.01; ***, *p* < 0.001; and ****, *p* < 0.0001).

## 3. Results

### 3.1. Screening of High β-Glucosidase Producing LAB

To select an LAB strain, which can effectively convert soy isoflavones into aglycones, *B. breve* CBT BR3 (BR3), *B. longum* CBT BG7 (BG7), *L. rhamnosus* CBT LR5 (LR5), *L. gasseri* CBT LGA1 (LGA1), and *L. lactis* subsp. *lactis* CBT SL6 (SL6) were compared for their β-glucosidase activities. These LAB strains were incubated in the S30 over 48 h at 37 °C, and their β-glucosidase activities were measured using whole cells without disrupting cells with *p*NPG as the substrate (Figure S1).

Among the tested strains, LGA1 exhibited the highest β-glucosidase activity. Specifically, the β-glucosidase activity of LGA1 was 3.06 U/mL after 2 h of incubation (highest level), whereas it decreased to 0.28 U/mL after 48 h of incubation (lowest level). The β-glucosidase activity of other strains after 1 h of incubation was as follows: BG7, 2.02 U/mL; LR5, 1.53 U/mL; BR3, 1.41 U/mL; and SL6, 0.33 U/mL. Overall, LGA1 exhibited the highest β-glucosidase activity against *p*NPG over the 48 h incubation period; therefore, it was selected as the LAB strain for fermenting S30 to convert soy isoflavones to aglycones.

### 3.2. Effect of S30+LGA1 on Estrogen Markers in MCF-7 Cells

During menopause, the estrogen level decreases [1]. Therefore, to investigate whether the combined S30 and LGA1, denoted as S30+LGA1, could increase the estrogen level, the mRNA expression of the genes of estrogen receptors, ESR1 and ESR2, and an estrogen-related gene of pS2 in MCF-7 cells [35,36] from human breast adenocarcinoma cell line, were analyzed.

Regarding the effect of treatment with S30+LGA1 and S30 and no treatment (control) on MCF-7 cells, S30+LGA1 exhibited significantly higher mRNA expression levels of ESR 1, ESR 2, and pS2 than the others (Figure S2). The mRNA expression level of ESR 1 with S30+LGA1 (2.62 ± 0.46, *p* < 0.0001) was 2.7-fold higher than that without treatment (1.00 ± 0.07). Additionally, the mRNA expression level of ESR 1 with S30+LGA1 (2.62 ± 0.46, *p* < 0.0001) was 2.9-fold higher than that with S30 (0.91 ± 0.17). Similarly, the mRNA expression level of ESR 2 with S30+LGA1 (3.19 ± 0.27, *p* < 0.0001) was 3.2-fold higher than that without treatment (1.01 ± 0.14). Moreover, the mRNA expression level of ESR 2 with S30+LGA1 (3.19 ± 0.27, *p* < 0.0001) was 4-fold higher than that with S30 (0.79 ± 0.28). Similarly, the mRNA expression level of pS2 with S30+LGA1 (3.88 ± 0.52, *p* < 0.0001) was 3.9-fold higher than that without treatment (1.00 ± 0.11) and 3.3-fold higher than that with S30 (1.17 ± 0.24). Thus, S30+LGA1 exhibited a significant effect on the upregulation of the genes of ESR1, ESR2, and pS2 in MCF-7 cells.

### 3.3. Effect of S30+LGA1 on Bone-Related Markers in MG-63 Cells

Bone loss, or osteoporosis, occurs during menopause [37]. The human osteosarcoma cell line (MG-63) was isolated from the bone of a patient with osteosarcoma [38]. To investigate whether treatment with S30+LGA1 could prevent or alleviate bone loss in menopause, the expressions of bone-related markers were analyzed using the MG-63 cells. Additionally, the mRNA expressions of ESR1 and ESR2 were analyzed (Figure S3).

The carboxylated form of OC binds calcium directly, thus concentrating in bone [39,40,41,42,43]. The mRNA expression of OC with S30+LGA1 (2.84 ± 0.31, *p* < 0.0001) was 2.8-fold higher than that without treatment (1.01 ± 0.12) (Figure S3A). The mRNA expression of OC with S30+LGA1 (2.84 ± 0.31, *p* < 0.001) was 2.2-fold higher than that with S30 (1.27 ± 0.39).

Osteoprotegerin (OPG) or osteoclastogenesis inhibitory factor regulates bone density [44]. The mRNA expression of OPG with S30+LGA1 (2.92 ± 0.29, *p* < 0.0001) was 3-fold higher than that without treatment (0.98 ± 0.23) and 2.8-fold higher than that with S30 (1.06 ± 0.27) (Figure S3B).

Bone morphogenetic protein-2 (BMP 2) induces bone generation and regeneration via extraskeletal and skeletal organogenesis [45]. The mRNA expression of BMP2 with S30+LGA1 (2.44 ± 0.21, *p* < 0.0001) was 2.4-fold higher than that without treatment (1.00 ± 0.08) (Figure S3C). The mRNA expression of BMP2 with S30+LGA1 (2.44 ± 0.21, *p* < 0.001) was 2.1-fold higher that with S30 (1.14 ± 0.32).

Morphogenetic protein 4 (BMP 4) is known to be involved in bone and cartilage development [46]. The mRNA expression of BMP4 with S30+LGA1 (3.57 ± 0.31, *p* < 0.0001) was 3.4-fold higher than that without treatment (1.05 ± 0.16) (Figure S3D). The mRNA expression of BMP4 with S30+LGA1 (3.57 ± 0.31, *p* < 0.0001) was 2.4-fold higher than that with S30 (1.49 ± 0.27).

Collagen type I (Col1) is the most abundant protein in mammals, contributing to 90% of the total organic component of bone matrix [47]. The mRNA expression of COL1 alpha 1 chain (COL1A1) with S30+LGA1 (4.15 ± 0.37, *p* < 0.0001) was 4.15-fold higher than that without treatment (1.00 ± 0.08) (Figure S3E). The mRNA expression of COL1A1 with S30+LGA1 (4.15 ± 0.37, *p* < 0.0001) was 3.6-fold higher than that with S30 (1.16 ± 0.28).

Alkaline phosphatase (ALP) contributes to bone growth and fracture healing [48]. The mRNA expression of ALP with S30+LGA1 (2.53 ± 0.33, *p* < 0.0001) was 2.5-fold higher than that without treatment (1.01 ± 0.12) (Figure S3F). The mRNA expression of ALP with S30+LGA1 (2.53 ± 0.33, *p* < 0.0001) was 2.3-fold higher than that with S30 (1.10 ± 0.22).

The mRNA expression of ESR1 with S30+LGA1 (3.64 ± 0.41, *p* < 0.0001) was 3.6-fold higher than that without treatment (1.01 ± 0.15). The mRNA expression of ESR1 with S30+LGA1 (3.64 ± 0.41, *p* < 0.0001) was 3.1-fold higher than that with S30 (1.19 ± 0.36) (Figure S3G). The mRNA expression of ESR2 with S30+LGA1 (3.04 ± 0.61, *p* < 0.001) was 3-fold higher than that without treatment (1.01 ± 0.14) (Figure S3F). The mRNA expression of ESR2 with S30+LGA1 (3.04 ± 0.61, *p* < 0.01) was 2.4-fold higher than that with S30 (1.25 ± 0.60).

### 3.4. Effect of S30+LGA1 on the Body Weight of OVX Rats

The effect of S30+LGA1 on the body weight of OVX rats was investigated (Figure 1). The body weight gain in the OVX group (178.7 ± 18.3 g) was significantly increased by 180% compared with that in the sham group (99.3 ± 16.1 g, *p* < 0.0001) (Figure 1B). However, the OVX+E2 (107.8 ± 9.9 g, *p* < 0.0001), OVX+LGA1 (116.0 ± 30.3 g, *p* < 0.0001), and OVX+S30+LGA1 (124.3 ± 5.2 g, *p* < 0.0001) groups exhibited significant decreases in body weight gain compared with the sham group. However, the OVX+S30 group did not exhibit any significant differences compared with the OVX group.

The serum level of 17β-estradiol in the OVX group (19.3 ± 9.0 pg/mL) was significantly decreased by 21% compared with that in the sham group (90.7 ± 19.5 pg/mL, *p* < 0.0001) (Figure 1C). However, the OVX+E2 group (89.0 ± 11.5 pg/mL, *p* < 0.0001) exhibited a significant increase in 17β-estradiol compared with the OVX group (19.3 ± 9.0 pg/mL). The other groups did not exhibit any significant differences compared with the OVX group.

### 3.5. Effect of S30+LGA1 on the Abdominal Fat Volume in OVX Rats

**Figure 2:**
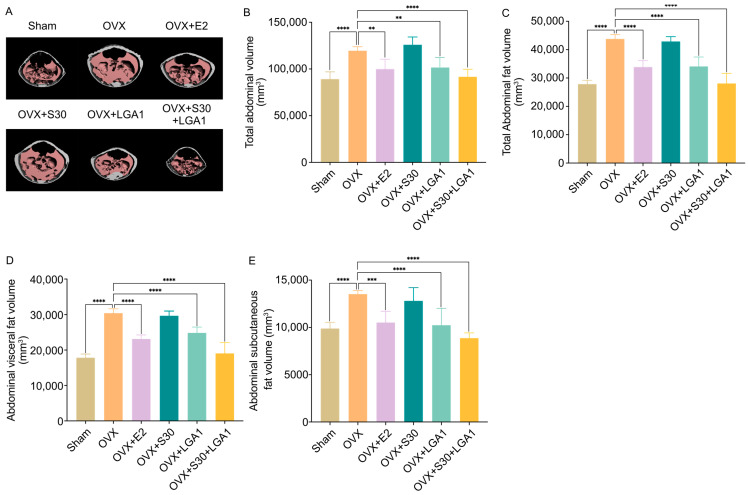
Effect of S30+LGA1 on abdominal fat volume in OVX rats evaluated using micro-CT. The OVX rats were administered S30+LGA1 for 8 weeks. (**A**) Micro-CT image of the abdominal fat area, (**B**) total abdominal volume, (**C**) total abdominal fat volume, (**D**) abdominal visceral fat volume, and (**E**) abdominal subcutaneous fat volume analyzed via micro-CT. Data are presented as the mean ± standard deviation (SD). ** *p* < 0.01, *** *p* < 0.001, **** *p* < 0.0001 compared with the OVX group. OVX, ovariectomized; E2, 17β-Estradiol; S30, soybean germ extract; LGA1, *Lactobacillus gasseri*.

The effect of S30+LGA1 on the abdominal fat volume in the OVX rats was investigated using micro-CT images. We observed significant increases in the abdominal fat volume in the OVX group compared with that in the sham group (Figure 2A). However, the abdominal fat volume in the OVX+S30+LGA1 group was decreased compared with that in the sham group. Regarding total abdominal volume, the OVX group exhibited a notable increase compared with the sham group. The total abdominal volume in the OVX group (119,525.1 ± 4281.1 mm^3^) was increased by 134% compared with that in the sham group (89,208.4 ± 7702.5 mm^3^, *p* < 0.0001) (Figure 2B). However, the OVX+E2 (99,930.9 ± 10473.7 mm^3^, *p* < 0.01), OVX+LGA1 (101,542.5 ± 10171.3 mm^3^, *p* < 0.01), and OVX+S30+LGA1 (91,633.2 ± 8074.9 mm^3^, *p* < 0.0001) groups exhibited significant decreases in the total abdominal volume compared with the OVX group. The other groups did not exhibit any significant differences compared with the OVX group.

Similarly, the total abdominal fat volume in the OVX group (43,794.4 ± 1509.7 mm^3^, *p* < 0.0001) was significantly increased by 157% compared with that in the sham group (27,844.4 ± 1313.2 mm^3^, *p* < 0.0001) (Figure 2C). However, the OVX+E2 (33,881.9 ± 2262.6 mm^3^, *p* < 0.0001), OVX+LGA1 (34,084.0 ± 3331.6 mm^3^, *p* < 0.0001), and OVX+S30+LGA1 (28,076.5 ± 3516.7 mm^3^, *p* < 0.0001) groups exhibited significant decreases in the total abdominal fat volume compared with the OVX group. The other groups did not exhibit any significant differences compared with the OVX group.

The abdominal visceral fat volume in the OVX group (30,411.8 ± 1189.2 mm^3^, *p* < 0.0001) was significantly increased by 170% compared with that in the sham group (17,836.0 ± 1037.3 mm^3^, *p* < 0.0001) (Figure 2D). However, the OVX+E2 (23,131.3 ± 1144.2 mm^3^, *p* < 0.0001), OVX+LGA1 (24,837.7 ± 1649.5 mm^3^, *p* < 0.0001), and OVX+S30+LGA1 (19,057.5 ± 3068.5 mm^3^, *p* < 0.0001) groups exhibited significant decreases in the abdominal visceral fat volume compared with the OVX group. The other groups did not exhibit any significant differences compared with the OVX group.

The abdominal subcutaneous fat volume in the OVX group (13,515.6 ± 370.6 mm^3^, *p* < 0.0001) was significantly increased by 137% compared with that in the sham group (9888.5 ± 651.9 mm^3^, *p* < 0.0001) (Figure 2E). However, the OVX+E2 (10,524.5 ± 1174.0 mm^3^, *p* < 0.0001), OVX+LGA1 (10,240.5 ± 1766.5 mm^3^, *p* < 0.0001), and OVX+S30+LGA1 (8875.8 ± 555.9 mm^3^, *p* < 0.0001) groups exhibited significant decreases compared with the OVX group. The other groups did not exhibit any significant differences compared with the OVX group.

### 3.6. Effect of S30+LGA1 on Uterine Weight and Tissues in OVX Rats

**Figure 3:**
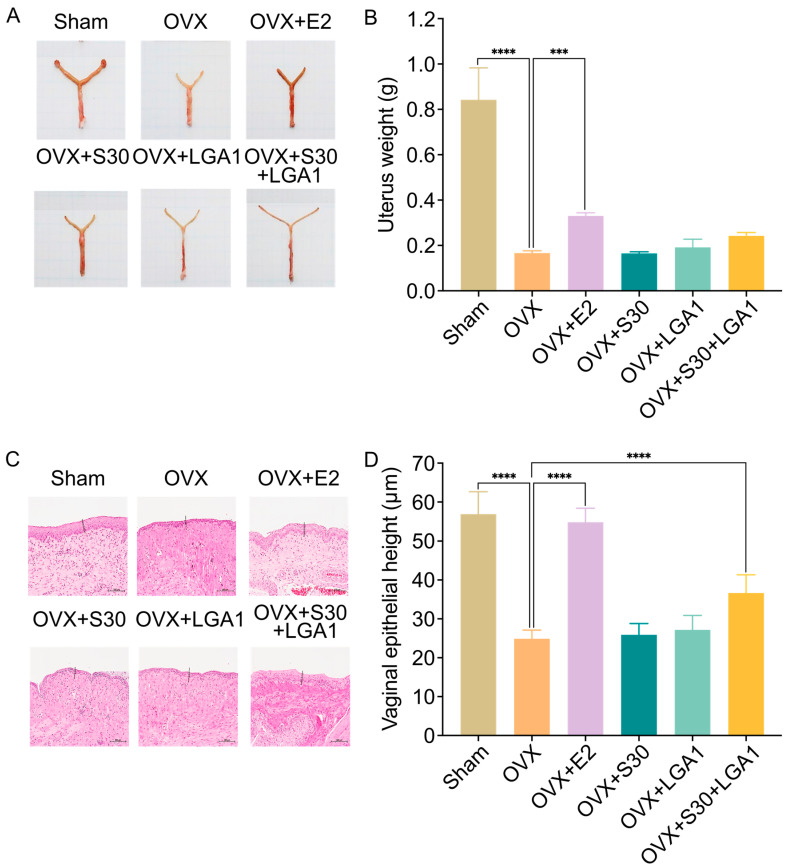
Effect of S30+LGA1 on uterine weight and tissues in OVX rats. (**A**) Representative images of rat uteri and (**B**) the weight of rat uteri. (**C**) Hematoxylin and eosin stained sections of the vaginal epithelium and (**D**) a graphical representation of the measured vaginal epithelial height. Data are presented as the mean ± standard deviation (SD). *** *p* < 0.001, **** *p* < 0.0001 compared with the OVX group. OVX, ovariectomized; E2, 17β-Estradiol; S30, soybean germ extract; LGA1, *Lactobacillus gasseri*.

To investigate the effect of S30+LGA1 on uterine weight and tissues in the OVX rats, the animals in the six study groups were euthanized, and the uterine weights were subsequently measured. The uterine weight was decreased in the OVX group compared with that in the sham group (Figure 3A). Moreover, the results revealed that the uterine weight in the OVX group (0.17 ± 0.01 g) was significantly lower than that in the sham group (0.84 ± 0.14 g, *p* < 0.0001), indicating the successful establishment of ovarian hormone loss (Figure 3B). The uterine weight in the OVX+ E2 group (0.33 ± 0.01 g, *p* < 0.001) was significantly increased compared with that in the OVX group (0.17 ± 0.01 g). The uterine weight in the other groups did not differ significantly from that in the OVX group.

Furthermore, significant differences were observed in the vaginal epithelial height among the groups. The effects on uterine histomorphology revealed that the sham and OVX groups exhibited tall columnar and low cuboidal epithelia, respectively (Figure 3C). However, in the OVX+E2 and OVX+S30+LGA1 groups, the uterine epithelial cells resembled those of the sham-operated group. The vaginal epithelial height of the OVX group (24.87 ± 2.23 μm) was significantly decreased compared with that in the sham group (56.87 ± 5.77 μm, *p* < 0.0001) (Figure 3D). However, the OVX+E2 (54.78 ± 3.60 μm, *p* < 0.0001) and OVX+S30+LGA1 (36.65 ± 4.67 μm, *p* < 0.0001) groups demonstrated marginal increases in epithelial thickness compared with the OVX group (24.87 ± 2.23 μm). The OVX+S30 and OVX+LGA1 groups did not exhibit any significant differences compared with the OVX group.

### 3.7. Effect of S30+LGA1 on Bone Losses in OVX Rats

**Figure 4:**
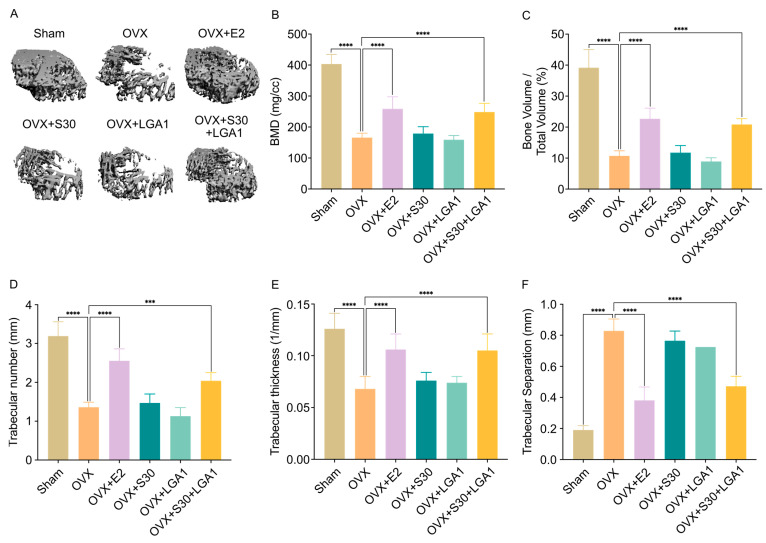
Effect of S30+LGA1 on the femur in OVX rats. (**A**) Micro-CT analysis of the left femur of OVX rats. (**B**) Bone mineral density (BMD), (**C**) trabecular bone volume (BV/TV), (**D**) trabecular number (Tb. N), (**E**) mean trabecular thickness (Tb. Th), (**F**) trabecular separation (Tb. Sp) were analyzed via micro-CT. Data are presented as the mean ± standard deviation (SD). *** *p* < 0.001, **** *p* < 0.0001 compared with the OVX group. OVX, ovariectomized; E2, 17β-Estradiol; S30, soybean germ extract; LGA1, *Lactobacillus gasseri*.

To investigate the effect of S30+LGA1 on ovariectomy-induced bone loss, micro-CT images of the proximal tibia were used (Figure 4A). The images clearly demonstrated a significant reduction in trabecular bones in the OVX rats. Additionally, various bone morphometric parameters were obtained through micro-CT analysis (Figure 4). The ANOVA results revealed that the OVX group exhibited significant decreases in BMD compared with the sham group (Figure 4B). The BMD in the OVX group (156.73 ± 14.33 mg/cc) was decreased by 39% compared with that in the sham group (403.42 ± 30.82 mg/cc, *p* < 0.0001). However, both the OVX+E2 (258.81 ± 38.82 mg/cc, *p* < 0.0001) and OVX+S30+LGA1 (248.33 ± 27.93 mg/cc, *p* < 0.0001) groups exhibited increases in BMD compared with the OVX group. The OVX+S30 and OVX+LGA1 groups did not exhibit any significant differences. Moreover, the bone volume fraction (BV/TV) ratio in the OVX group (10.72 ± 1.63%) was significantly decreased by 27% compared with that in the sham group (39.16 ± 5.87%, *p* < 0.0001) (Figure 4C). However, both the OVX+E2 (22.67 ± 3.43%) and OVX+S30+LGA1 (20.85 ± 1.89%) groups exhibited increases compared with the OVX group. The OVX+S30 and OVX+LGA1 groups did not exhibit any significant differences.

Similarly, other bone parameters, such as the trabecular number (Tb. N) and trabecular thickness (Tb. Th), were significantly increased following supplementation with E2 and S30+LGA1 (Figure 4D,E). However, the Tb. N and Tb. Th of the OVX+S30 and OVX+LGA1 groups did not differ significantly compared with those in the OVX group. Trabecular separations (Tb. Sp) were significantly increased in the OVX group (0.828 ± 0.077 mm) compared with those in the sham group (0.190 ± 0.028 mm, *p* < 0.0001) (Figure 4F). The Tb. Sp of the OVX+E2 (0.381 ± 0.086 mm, *p* < 0.0001) and OVX+S30+LGA1 (0.472 ± 0.064 mm, *p* < 0.0001) groups exhibited a significant decrease, while the Tb. Sp of the OVX+S30 and OVX+LGA1 groups did not differ significantly compared with those in the OVX group. Overall, these results suggest the positive impact of S30+LGA1 on bone health, as evidenced by the improved BMD and other bone morphometric parameters.

### 3.8. Effect of S30+LGA1 on the Bone-Related Biochemical Markers in OVX Rats

**Figure 5:**
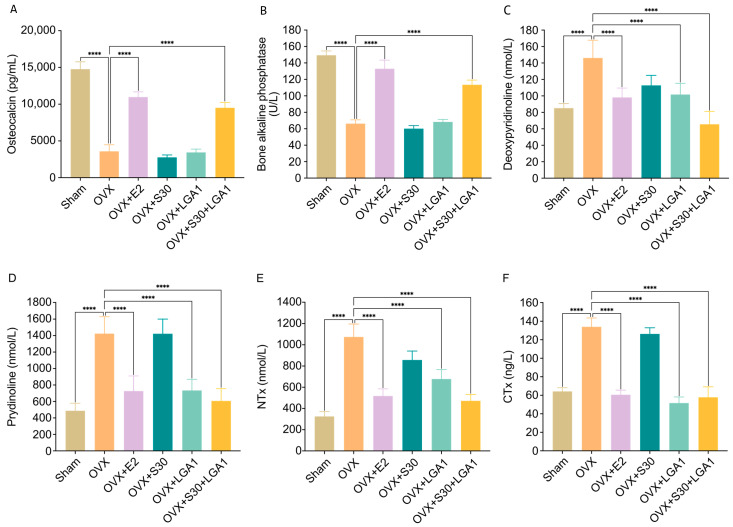
Effect of S30+LGA1 on bone-related biochemical markers in OVX rats. The levels of (**A**) osteocalcin and (**B**) bone alkaline phosphatase (B-ALP) are measured as markers of bone formation. The levels of (**C**) deoxypyridinoline, (**D**) prydinoline, (**E**) NTx, (**F**) CT_X_ are measured as markers of bone resorption. Data are presented as the mean ± standard deviation (SD). **** *p* < 0.0001 compared with the OVX group. OVX, ovariectomized; E2, 17β-Estradiol; S30, soybean germ extract; LGA1, *Lactobacillus gasseri*; NT_X_, amino-terminal cross-linked telopeptide of collagen; CT_X_, carboxy-terminal cross-linked telopeptide of collagen.

The effects of S30+LGA1 on the bone-related biochemical markers, such as OC and ALP, and bone resorption markers, including deoxypyridinoline (DPD), pyridinoline (PYD), amino-terminal cross-linked telopeptide of collagen (NTX), and carboxy-terminal cross-linked telopeptide of collagen (CTX), were investigated (Figure 5).

Regarding bone formation markers, the serum OC level in the OVX group (3586.4 ± 894.5 pg/mL) was significantly decreased by 24% compared with that in the sham-operated group (14764.4 ± 1002.6 pg/mL, *p* < 0.0001) (Figure 5A). However, both the OVX+E2 (10978.1 ± 702.0 pg/mL, *p* < 0.0001) and OVX+S30+LGA1 (9510.8 ± 725.6 pg/mL, *p* < 0.0001) groups exhibited significant increases in OC levels compared with the OVX group. The OVX+S30 and OVX+LGA1 groups did not exhibit any significant differences compared with the OVX group.

Similarly, the serum ALP level in the OVX group (66.2 ± 4.7 U/L) was significantly decreased by 44% compared with that in the sham group (149.4 ± 5.2 U/L, *p* < 0.0001) (Figure 5B). However, both the OVX+E2 (133.0 ± 10.5 U/L, *p* < 0.0001) and OVX+S30+LGA1 (113.5 ± 5.7 U/L, *p* < 0.0001) groups exhibited significant increases in ALP levels compared with the OVX group. The OVX+S30 and OVX+LGA1 groups did not exhibit any significant differences compared with the OVX group.

Regarding bone resorption markers, the serum level of DPD in the OVX group (146.1 ± 21.5 nmol/L) was significantly increased by 171.5% compared with that in the sham group (85.2 ± 5.5 nmol/L, *p* < 0.0001) (Figure 5C). However, the OVX+E2 (98.2 ± 11.3 nmol/L, *p* < 0.0001), OVX+LGA1 (101.7 ± 13.6 nmol/L, *p* < 0.0001), and OVX+S30+LGA1 (65.5 ± 15.7 nmol/L, *p* < 0.0001) groups exhibited significant decreases in DPD level compared with the OVX group. The OVX+S30 group did not exhibit any significant differences compared with the OVX group.

Similarly, the serum level of PYD in the OVX group (1423.7 ± 203.7 nmol/L) was significantly increased by 171.5% compared with that in the sham group (487 ± 91.2 nmol/L, *p* < 0.0001) (Figure 5D). However, the OVX+E2 (727 ± 183.5 nmol/L, *p* < 0.0001), OVX+LGA1 (733.7 ± 135.3 nmol/L, *p* < 0.0001), and OVX+S30+LGA1 (607 ± 149.7 nmol/L, *p* < 0.0001) groups exhibited significant decreases in PYD level compared with the OVX group. The OVX+S30 group did not exhibit any significant differences compared with the OVX group.

The NT_X_ level in the OVX group (1074.6 ± 121.2 nmol/L) was significantly increased by 330.7% compared with that in the sham group (324.9 ± 45.8 nmol/L, *p* < 0.0001) (Figure 5E). However, the OVX+E2 (518.2 ± 66.7 nmol/L, *p* < 0.0001), OVX+LGA1 (677.7 ± 89.0 nmol/L, *p* < 0.0001), and OVX+S30+LGA1 (472.1 ± 59.8 nmol/L, *p* < 0.0001) groups exhibited significant decreases in NTX level compared with the OVX group. The OVX+S30 group did not exhibit any significant differences compared with the OVX group.

The CT_X_ level in the OVX group (134.0 ± 9.5 ng/L) was significantly increased by 208.4% compared with that in the sham group (64.3 ± 4.0 ng/L, *p* < 0.0001) (Figure 5F). However, the OVX+E2 (60.6 ± 5.0 ng/L, *p* < 0.0001), OVX+LGA1 (51.7 ± 6.5 ng/L, *p* < 0.0001), and OVX+S30+LGA1 (57.9 ± 11.3 ng/L, *p* < 0.0001) groups exhibited significant decreases in CT_X_ level compared with the OVX group. The OVX+S30 group did not exhibit any significant differences compared with the OVX group. Overall, these results indicate the potential beneficial effects of S30+LGA1 on bone health, as evidenced by its influence on bone-related biochemical markers.

### 3.9. Effect of S30+LGA1 on Vascular Homeostasis in OVX Rats

**Figure 6:**
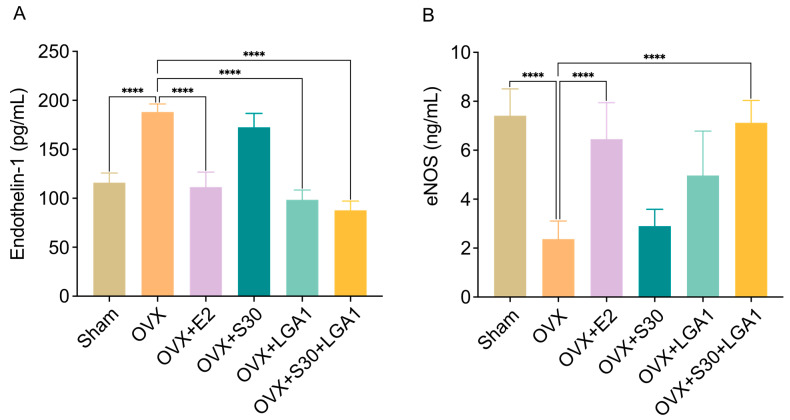
Effect of S30+LGA1 on vascular metabolism marker levels in the serum of OVX rats. (**A**) Endothelin-1 (ET-1) is measured as a serum marker of vasoconstriction. (**B**) Endothelial nitric oxide synthase (eNOS) is measured as a serum marker of vasodilation. Data are presented as the mean ± standard deviation (SD). **** *p* < 0.0001 compared with the OVX group. OVX, ovariectomized; E2, 17β-Estradiol; S30, soybean germ extract; LGA1, *Lactobacillus gasseri*.

The effect of S30+LGA1 on vascular homeostasis in OVX rats was investigated (Figure 6). The serum levels of endothelin-1 (ET-1) and endothelial nitric oxide synthase (eNOS) were measured using ELISA kits. The ET-1 level in the OVX group (188.1 ± 8.2 pg/mL) was significantly increased compared with that in the sham group (115.9 ± 9.8 pg/mL, *p* < 0.0001) (Figure 6A). However, the OVX+E2 (111.4 ± 15.3 pg/mL, *p* < 0.0001), OVX+LGA1 (98.4 ± 10.0 pg/mL, *p* < 0.0001), and OVX+S30+LGA1 (87.7 ± 9.5 pg/mL, *p* < 0.0001) groups exhibited significant decreases in ET-1 level compared with the sham group. The OVX+S30 group did not exhibit any significant differences compared with the OVX group.

The eNOS level in the OVX group (2.4 ± 0.7 ng/mL) was significantly decreased compared with that in the sham group (7.4 ± 1.1 pg/mL, *p* < 0.001) (Figure 6B). However, the OVX+E2 (6.5 ± 1.5 pg/mL, *p* < 0.001) and OVX+S30+LGA1 (7.1 ± 0.9 pg/mL, *p* < 0.001) groups exhibited significant increases in eNOS level compared with the sham group. The OVX+S30 and OVX+LGA1 groups did not exhibit any significant differences compared with the OVX group.

### 3.10. Effect of S30+LGA1 on Serotonin and Norepinephrine Levels in OVX Rats

**Figure 7:**
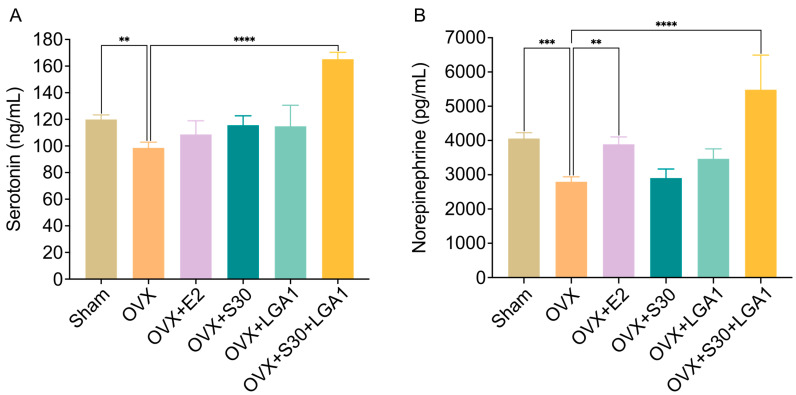
Effect of S30+LGA1 on serotonin and norepinephrine levels in the serum of OVX rats. (**A**) Serotonin and (**B**) norepinephrine levels are measured using ELISAs. Data are presented as the mean ± standard deviation (SD). ** *p* < 0.01, *** *p* < 0.001, **** *p* < 0.0001 compared with the OVX group. OVX, ovariectomized; E2, 17β-Estradiol; S30, soybean germ extract; LGA1, *Lactobacillus gasseri*.

To assess the effects of S30+LGA1 on neurotransmitters in OVX rats, the serum levels of serotonin and norepinephrine were measured. The serotonin level in the OVX group (98.5 ± 4.3 ng/mL) was significantly decreased compared with that in the sham group (119.9 ± 3.4 ng/mL, *p* < 0.01) (Figure 7A). However, the OVX+S30+LGA1 (165.2 ± 5.2 ng/mL, *p* < 0.0001) group exhibited a significant increase in serotonin level compared with the sham group. The OVX+E2, OVX+S30, and OVX+LGA1 groups did not exhibit any significant differences compared with the OVX group.

Similarly, the serum levels of norepinephrine in the OVX group (2795.8 ± 143.5 pg/mL) were significantly decreased compared with those in the sham group (4058.3 ± 170.8 pg/mL, *p* < 0.001) (Figure 7B). However, the OVX+E2 group (3891.7 ± 211.4 pg/mL, *p* < 0.01) exhibited an increase in norepinephrine level compared with the sham group. The OVX+S30+LGA1 group (5483.3 ± 1007.8 pg/mL, *p* < 0.0001) exhibited a significant increase in norepinephrine level compared with the sham group. However, the OVX+S30 and OVX+LGA1 groups did not exhibit any significant differences compared with the OVX group.

### 3.11. Effect of S30+LGA1 on Lipid Profile and Liver Enzymes, Albumin, and Calcium Levels in OVX Rats

The effects of S30+LGA1 on the lipid profile and AST, ALT, albumin, and calcium levels in the OVX rats were investigated. ELISA kits were used to measure the serum concentrations of these markers.

**Figure 8:**
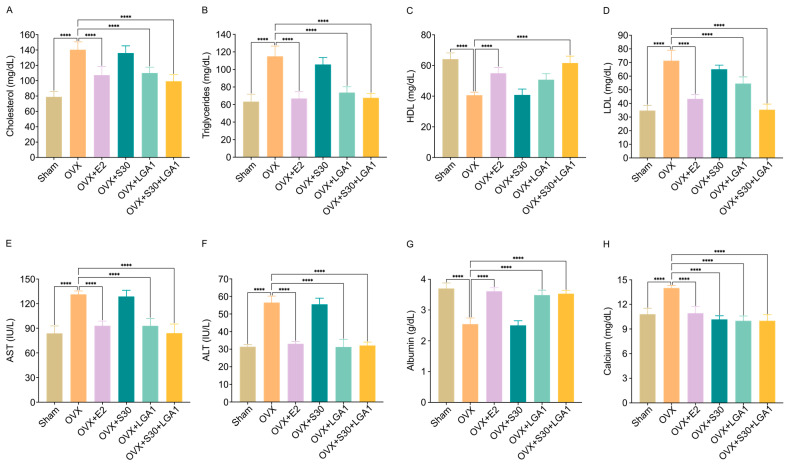
Effect of S30+LGA1 on lipid profile and AST, ALT, albumin, and calcium levels in the serum of OVX rats. (**A**) Cholesterol and (**B**) triglycerides, (**C**) HDL, (**D**) LDL, (**E**) AST, (**F**) ALT, (**G**) albumin, and (**H**) calcium levels were measured using ELISA. Data are presented as the mean ± standard deviation (SD). **** *p* < 0.0001 compared with the OVX group. OVX, ovariectomized; E2, 17β-Estradiol; S30, soybean germ extract; LGA1, *Lactobacillus gasseri*; HDL, high-density lipoprotein; LDL, low-density lipoprotein; AST, aspartate aminotransferase; ALT, alanine transaminase.

The serum cholesterol level in the OVX group (140.3 ± 10.2 mg/dL) was significantly increased compared with that in the sham group (79 ± 7.0 mg/dL, *p* < 0.01) (Figure 8A). However, the OVX+E2 (107.3 ± 11.0 mg/dL, *p* < 0.01), OVX+LGA1 (110 ± 7.6 mg/dL, *p* < 0.01), and OVX+S30+LGA1 (99.3 ± 8.5 mg/dL, *p* < 0.01) groups exhibited significant decreases in cholesterol level compared with the OVX group. The OVX+S30 group did not exhibit any significant differences compared with the OVX group.

Similarly, the serum triglycerides level in the OVX group (115 ± 11.7 mg/dL) was significantly decreased compared with that in the sham group (63.3 ± 8.4 mg/dL, *p* < 0.0001) (Figure 8B). However, the OVX+E2 (67 ± 7.7 mg/dL, *p* < 0.0001), OVX+LGA1 (73.7 ± 6.6 mg/dL, *p* < 0.0001), and OVX+S30+LGA1 (67.7 ± 5.0 mg/dL, *p* < 0.0001) groups exhibited significant increases in triglycerides level compared with the OVX group. The OVX+S30 group did not exhibit any significant differences compared with the OVX group.

The serum HDL level in the OVX group (40.7 ± 1.9 mg/dL) was significantly decreased compared with that in the sham group (64.2 ± 4.0 mg/dL, *p* < 0.0001) (Figure 8C). However, the OVX+E2 (55.0 ± 3.7 mg/dL, *p* < 0.0001) and OVX+S30+LGA1 (61.6 ± 4.4 mg/dL, *p* < 0.0001) groups exhibited significant increases in HDL level compared with the OVX group. The OVX+S30 and OVX+LGA1 groups did not exhibit any significant differences compared with the OVX group.

The serum LDL level in the OVX group (71.32 ± 7.7 mg/dL) was significantly increased compared with that in the sham group (34.8 ± 3.8 mg/dL, *p* < 0.0001) (Figure 8D). However, the OVX+E2 (43.4 ± 3.2 mg/dL, *p* < 0.0001), OVX+LGA1 (54.6 ± 4.8 mg/dL, *p* < 0.0001), and OVX+S30+LGA1 (35.4 ± 4.1 mg/dL, *p* < 0.0001) groups exhibited significant decreases in LDL level compared with the OVX group. The OVX+S30 group did not exhibit any significant differences compared with the OVX group.

The serum AST level in the OVX group (131.3 ± 4.2 IU/L) was significantly increased compared with that in the sham group (83.8 ± 9.0 IU/L, *p* < 0.0001) (Figure 8E). However, the OVX+E2 (93.1 ± 5.6 IU/L, *p* < 0.0001), OVX+LGA1 (92.9 ± 8.9 IU/L, *p* < 0.0001), and OVX+S30+LGA1 (84.1 ± 11.1 IU/L, *p* < 0.0001) groups exhibited significant decreases in AST level compared with the OVX group. The OVX+S30 group did not exhibit any significant differences compared with the OVX group.

The serum ALT level in the OVX group (56.5 ± 3.7 IU/L) was significantly increased compared with that in the sham group (31.4 ± 1.2 IU/L, *p* < 0.0001) (Figure 8F). However, the OVX+E2 (33.1 ± 1.4 IU/L, *p* < 0.0001), OVX+LGA1 (31.3 ± 4.4 IU/L, *p* < 0.0001), and OVX+S30+LGA1 (32.1 ± 1.9 IU/L, *p* < 0.0001) groups exhibited significant decreases in AST level compared with the OVX group. The OVX+S30 group did not exhibit any significant differences compared with the OVX group.

The serum albumin level in the OVX group (2.5 ± 0.2 g/dL) was significantly decreased compared with that in the sham group (3.7 ± 0.2 g/dL, *p* < 0.0001) (Figure 8G). However, the OVX+E2 (3.6 ± 0.1 g/dL, *p* < 0.0001), OVX+LGA1 (3.5 ± 0.2 g/dL, *p* < 0.0001), and OVX+S30+LGA1 (3.5 ± 0.1 g/dL, *p* < 0.0001) groups exhibited significant increases in albumin level compared with the OVX group. The OVX+S30 group did not exhibit any significant differences compared with the OVX group.

The serum calcium level in the OVX group (14.0 ± 0.3 mg/dL) was significantly increased compared with that in the sham group (10.8 ± 0.7 mg/dL, *p* < 0.0001) (Figure 8H). However, the OVX+E2 (10.9 ± 0.8 mg/dL, *p* < 0.0001), OVX+S30 (10.2 ± 0.4 mg/dL, *p* < 0.0001), OVX+LGA1 (10.0 ± 0.6 mg/dL, *p* < 0.0001), and OVX+S30+LGA1 (10.0 ± 0.7 mg/dL, *p* < 0.0001) groups exhibited significant decreases in calcium level compared with the OVX group.

## 4. Discussion

Conventionally, HRT supplements are used to relieve menopausal symptoms [49,50]. However, these have several side effects [51,52,53,54]. Plant-derived therapies can potentially offer effective treatment with fewer side effects [55]. Therefore, the exploration of alternative therapies for managing menopausal symptoms has garnered considerable interest. The phytoestrogens found in soybeans present one such alternative therapy. Phytoestrogens have estrogen-like effects on the body [56], and their ability to bind to estrogen receptors and mimic the actions of estrogen provides some relief from menopausal symptoms [57]. Soy-based products and their isoflavones have gained popularity as a safer and potentially effective option for addressing hormonal-imbalance-related health conditions [58,59]. In the present study, we investigated the effects of S30+LGA1—a soy-derived compound and probiotic combination—on estrogen markers, abdominal fat volume, and serum markers related to bone health and vascular homeostasis in OVX rat models.

Several probiotic LAB strains have demonstrated potential in converting isoflavone glucosides to aglycones [19]. Isoflavone aglycones have a positive impact on menopausal symptoms, including depression, obesity, and osteoporosis [60,61,62]. In our study, the screening of probiotics for their β-glucosidase activity revealed significant differences among the tested LAB strains. Notably, LGA1 exhibited the highest and most efficient β-glucosidase activity throughout the 48-h fermentation period in S30 solution, indicating that LGA1 is highly capable of hydrolyzing isoflavone glucosides into their active aglycone forms and enhancing the bioavailability of isoflavone aglycones from soy-based products. Therefore, assessing β-glucosidase activity is crucial, as it plays a key role in this conversion [63].

Estrogen deficiency causes rapid bone loss in post-menopausal women [64]. Estrogen is a key regulator of bone remodeling [65]. The results of our study on the MCF-7 and MG-63 cells demonstrated the significant effects of S30+LGA1 treatment on the expression of estrogen and bone-related genes. In the MCF-7 cells, S30+LGA1 upregulated the expression of ESR 1, ESR 2, and pS2, suggesting that S30+LGA1 may positively influence estrogen signaling pathways in MCF-7 cells. Similarly, in MG-63 cells, S30+LGA1 significantly increased the expressions of OC, OPG, BMP 2, BMP 4, COL1A1, ALP, ESR 1, and ESR 2. These findings suggest that S30+LGA1 may promote bone-related processes in MG-63 cells. The S30+LGA1 combination may synergistically promote bone health and mitigate menopause-related bone issues owing to the β-glucosidase activity of *L. gasseri*, which enhances the bioavailability of isoflavones from soy-based products [66].

This study evaluated the effect of S30+LGA1 on various menopause-related aspects in OVX rat models. Menopausal women commonly tend to gain weight. Estrogen regulates body fat production because estrogen suppresses lipoprotein lipase at both mRNA and protein levels [67,68,69]. Therefore, estrogen deficiency is associated with an increase in adipocytes [69]. The OVX rats exhibited a significant increase in body weight, which is consistent with findings in previous studies, namely that ovariectomy can lead to weight gain due to hormonal changes and altered metabolism [70]. A diet supplemented with S30+LGA1 may have had beneficial effects in ameliorating the weight gain in the treated group. Additionally, the increase in serum 17β-estradiol levels in the OVX+E2 group indicates the potential estrogenic effects of the treatment. The serum 17β-estradiol levels in the OVX group were significantly decreased, as expected, because the ovaries were the primary source of estrogen production [70]. The OVX+E2 group exhibited a significant increase in 17β-estradiol levels compared with the OVX group. However, the other groups, including OVX+S30+LGA1, did not exhibit a significant difference in 17β-estradiol levels compared with the OVX group, suggesting that the effects of S30+LGA1 on estrogen markers may not be mediated through direct estrogenic activity.

The results of our study demonstrate the potential of S30+LGA1 in reducing abdominal fat volume. The administration of S30+LGA1 led to a notable reduction in total abdominal fat volume in the treated groups. Furthermore, we observed significant reductions in abdominal visceral and subcutaneous fat volumes in the OVX+E2, OVX+LGA1, and OVX+S30+LGA1 groups compared with the OVX group. These results provide strong evidence of the potential anti-obesity effects of S30+LGA1. Our findings are in line with previous studies, which investigated the effects of estradiol and soybean isoflavones and aglycones on fat metabolism [71]. These compounds can inhibit abnormal lipid metabolism and fat synthesis [71]. Soybean isoflavones, such as genistein and daidzein, reduce adipocyte size and promote fat oxidation, which may contribute to the reduction in abdominal fat volume, similar to that observed in our study [71,72]. Moreover, soy-based compounds have positive effects on adipose tissue metabolism and reduce inflammation and oxidative stress in the adipose tissue [73,74]. These beneficial effects may have had a role in the reduction in abdominal fat volume in the OVX rats treated with S30+LGA1. These findings suggest that S30+LGA1 may help mitigate post-menopausal abdominal fat accumulation.

In the OVX rats, estradiol and soybean isoflavones and aglycones were found to alleviate uterine atrophy caused by the significantly decreased estrogen levels [75]. The most significant effects were observed with the combination of estradiol and isoflavones and aglycones [75]. In this study, we investigated the effects of S30+LGA1 on uterine weight and tissues in the OVX rats (Figure 3). The data indicate that both the uterine weight and vaginal epithelial height were marginally increased in both the OVX+E2 and OVX+S30+LGA1 groups compared to the OVX group. A previous study demonstrated similar results, revealing that the post-ovariectomy uterine weight was lower in OVX rats compared to that in normal female rats, indicating gradual uterine atrophy [76]. These results suggest the potentially beneficial impact of S30+LGA1 on uterine health.

In this study, we investigated the effects of S30+LGA1 on bone loss in OVX rats using micro-CT images. In the sham group, no significant changes were observed in the five bone indices. However, the OVX group exhibited significant decreases in BMD, BV/TV, Tb. N, and Tb. Th, and an increase in Tb. Sp (Figure 4), indicating the development of osteoporosis. Bilateral ovariectomy in rats reduced estrogen levels, inhibiting estrogen receptor expression on osteoblasts and osteoclasts [77,78]. This disruption led to hyperfunction of osteoclasts, thereby increasing bone resorption and promoting osteoporosis [77,78]. In our study, both the OVX+E2 and OVX+S30+LGA1 groups exhibited a significant increase in bone indices compared with those in the OVX group. Estradiol improves menopausal osteoporosis; however, long-term use may lead to an increased risk of cardiovascular events, breast hyperplasia, and uterine fibroids [79]. Our results indicate that in post-menopausal women, S30+LGA1 may potentially prevent bone loss and reduce fracture risk by inhibiting osteoclast activity.

Regarding serum bone markers, the OVX group exhibited decreased levels of OC and ALP, which are the markers of bone formation, and increased levels of DPD, PYD, NT_X_, and CT_X_, which are the markers of bone resorption, compared with the sham group. Physiological bone changes are typically triggered by decreased estrogen levels after ovariectomy [80]. Osteopenia occurs when bone turnover accelerates, leading to increased resorption surpassing bone formation [81]. However, the OVX+E2 and OVX+S30+LGA1 groups exhibited significant improvement in bone formation and reduction in bone resorption markers compared with the OVX group. These findings suggest that S30+LGA1 may serve as an alternative for HRT for preventing osteoporosis.

Previous studies demonstrated that estrogen supplementation may protect against hypertension by activating vasodilation pathways, such as the nitric oxide (NO) system, and inhibiting vasoconstricting systems, such as the endothelin (ET) system [82,83,84,85,86,87,88,89]. Regarding vascular homeostasis markers, the OVX group in our study exhibited increased and decreased levels of ET-1 and eNOS, respectively. However, the OVX+E2 and OVX+S30+LGA1 groups exhibited significant decreases in ET-1 levels and increases in eNOS levels compared with the OVX group, indicating potential improvements in vascular function. These effects may contribute to improved cardiovascular health and reduced risk of cardiovascular diseases in menopausal women. However, while our vascular homeostasis study provided promising results regarding the effects of S30+LGA1 on vascular function in menopausal conditions of OVX rats, the measurement of skin tail temperature was not included in this study. This measure is used as a surrogate for assessing vasomotor instability, which is a hallmark of menopause [90]. To gain a more comprehensive understanding of the potential benefits of S30+LGA1 in managing vasomotor symptoms, it needs to be studied in clinical trials.

Estrogen and phytoestrogen treatment can increase serotonin and norepinephrine levels, leading to the alleviation of depressive symptoms [91,92,93]. In our study, we investigated the effects of S30+LGA1 on the serum levels of serotonin and norepinephrine, which are mood-regulating neurotransmitters. The OVX group exhibited decreased levels of serotonin and norepinephrine, which may be associated with mood disturbances and depression often observed in menopausal women. Notably, the OVX+S30+LGA1 group demonstrated significant increases in both serotonin and norepinephrine levels compared with the OVX group, suggesting the potential mood-enhancing effects of S30+LGA1.

Menopausal women often experience alterations in lipid metabolism and liver function, which can lead to increased risks of cardiovascular diseases and other health complications [94,95]. Therefore, understanding the impact of S30+LGA1 on these parameters is crucial for assessing its potential as an alternative therapy for managing menopausal symptoms. In this study, we further investigated the effects of S30+LGA1 on serum lipid profile and liver enzymes, albumin, and calcium levels in OVX rats. We observed significant changes in serum lipid profiles in the OVX rats. The OVX group exhibited elevated levels of cholesterol, triglycerides, and LDL and decreased levels of HDL. These changes are consistent with those reported in a previous study, namely that estrogen deficiency contributes to dyslipidemia and increased cardiovascular risk in menopausal women [96]. The treatment with S30+LGA1 led to notable improvements in lipid profiles. We observed decreased cholesterol, triglycerides, and LDL levels and increased HDL levels in the OVX+S30+LGA1 group, similar to the effects observed in the OVX+E2 group. These results suggest that S30+LGA1 supplementation may help restore lipid balance and reduce the risk of menopause-related cardiovascular complications.

Liver enzymes, such as AST and ALT, are essential indicators of liver function [97]. Elevated levels of these enzymes in the OVX group indicated liver damage, possibly due to the altered lipid metabolism and hormonal changes after ovariectomy [98]. However, treatment with S30+LGA1 significantly reduced AST and ALT levels in the OVX rats. These findings suggest that S30+LGA1 may possess hepatoprotective properties and support liver health during menopause. Albumin, a crucial liver-synthesized protein, helps maintain oncotic pressure and transport various substances in the blood [99]. The decreased level of albumin in the OVX group indicated impaired liver function and consequential reduced albumin synthesis. However, treatment with S30+LGA1 restored albumin levels, indicating its potential positive effect on liver function and overall health. Calcium is an essential mineral, which plays a vital role in bone health and various physiological processes [100]. Notably, treatment with S30+LGA1 significantly increased calcium levels, suggesting its potential role in mitigating bone loss and improving calcium homeostasis during menopause.

In summary, our study investigated the effects of S30+LGA1—a soy-derived compound combined with a probiotic LAB—on menopausal conditions in OVX rats. Supplementation with S30+LGA1 yielded promising results, such as improved lipid profile and hepato- and osteo-protective effects. Additionally, S30+LGA1 demonstrated a positive impact on uterine health and mood regulation. These findings suggest that S30+LGA1 may be a natural, effective, and beneficial intervention for menopausal symptoms. To further assess the efficacy of S30+LGA1, a clinical trial needs to be conducted in the future.

## 5. Conclusions

Our study highlights the potential benefits of S30+LGA1 for menopausal symptoms. S30+LGA1 exhibited positive effects on various aspects, including body weight, uterine and bone health, vascular markers, neurotransmitter levels, lipid profile, and liver enzymes. These findings suggest that S30+LGA1 could be a promising intervention for alleviating menopausal symptoms and addressing various menopause-related health aspects in the OVX model. Therefore, S30+LGA1 can be recommended as a soy-based diet as an alternative or supplement to HRT in menopausal women.

## Data Availability

The datasets used and analyzed in this study are available from the corresponding author on reasonable request.

## Supplementary Materials

The following supporting information can be downloaded at: https://www.mdpi.com/article/10.3390/nu15204485/s1, Figure S1: β-glucosidase activity of probiotics in S30 at 37 °C for 48 h; Figure S2: Effects of S30+LGA1 on estrogen-related mRNA expression level in MCF-7 cells; Figure S3: Effects of S30+LGA1 on estrogen and bone-related mRNA expression level in MG-63; Table S1: Primer sequences for real-time RT-PCR analysis of mRNA quantification.
