# Supplementary material for: Anti-Menopausal Effect of Soybean Germ Extract and Lactobacillus gasseri in the Ovariectomized Rat Model
Source: Nutrients. 2023 Oct 23;15(20):4485. doi: 10.3390/nu15204485 (PMC10609938; doi:10.3390/nu15204485)
Supplement: Supplementary file 1 [file nutrients-15-04485-s001.zip › nutrients-2658463-supplementary.docx]

**
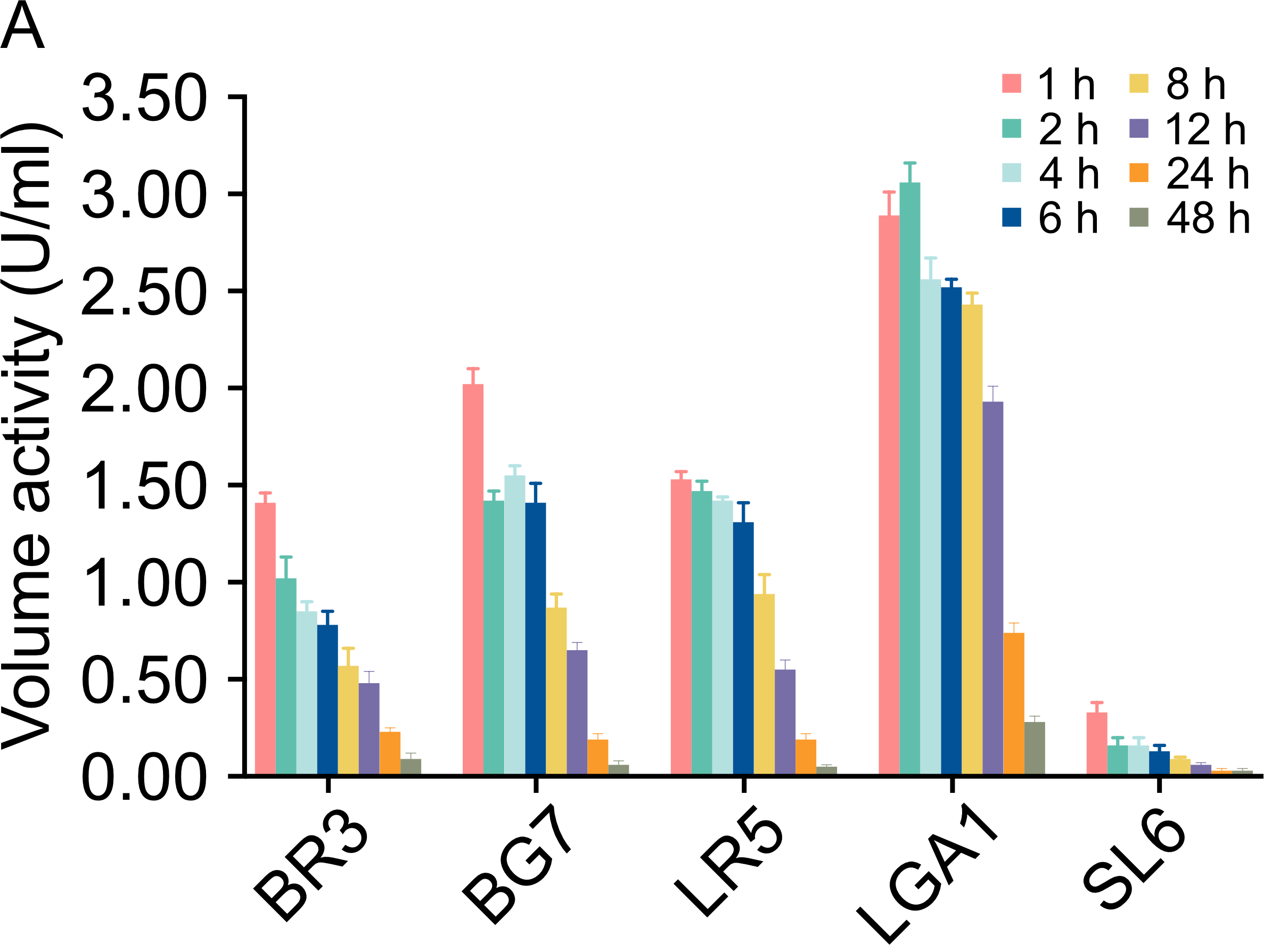
**

**Figure S1.** β-glucosidase activity of probiotics in S30 at 37°C for 48 h. One unit of enzyme activity was defined as the amount of β-glucosidase that released 1 μmol of *p*-nitrophenol from the *p*NPG substrate per mL per min under the assay conditions. *p*NPG, *p*-nitrophenyl-β-D-glucopyranoside; S30, soybean germ extract; LGA1, *Lactobacillus gasseri*; BR3*, Bifidobacterium breve*; BG7*, Bifidobacterium longum;* LR5*, Lactobacillus rhamnosus*; SL6*, Lactococcus lactis* subsp. lactis

**
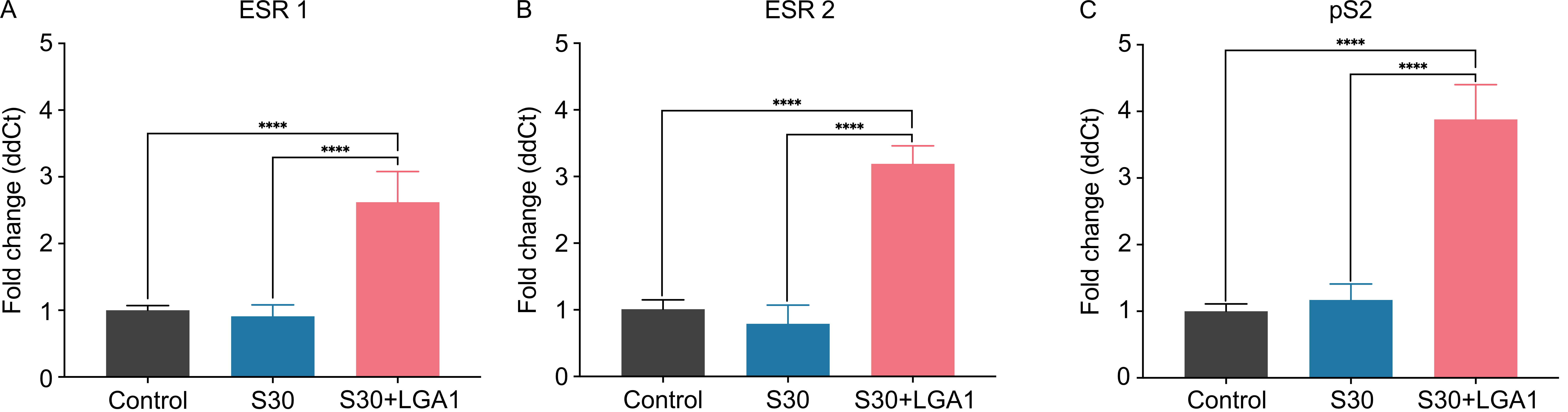
**

**Figure S2.** Effect of S30+LGA1 on estrogen-related mRNA expression level in MCF-7 cells. The mRNA expression of **(A)** ESR1, **(B)** ESR2, and (C) pS2 are evaluated. After extracting total RNA from the cells, the mRNA levels are quantified using real-time PCR. The obtained values are normalized based on the expression level of β-actin. Data are presented as the mean ± standard deviations (SD). *****p* < 0.0001 when compared with the control group. S30, soybean germ extract; LGA1, *Lactobacillus gasseri*; ESR, estrogen receptor


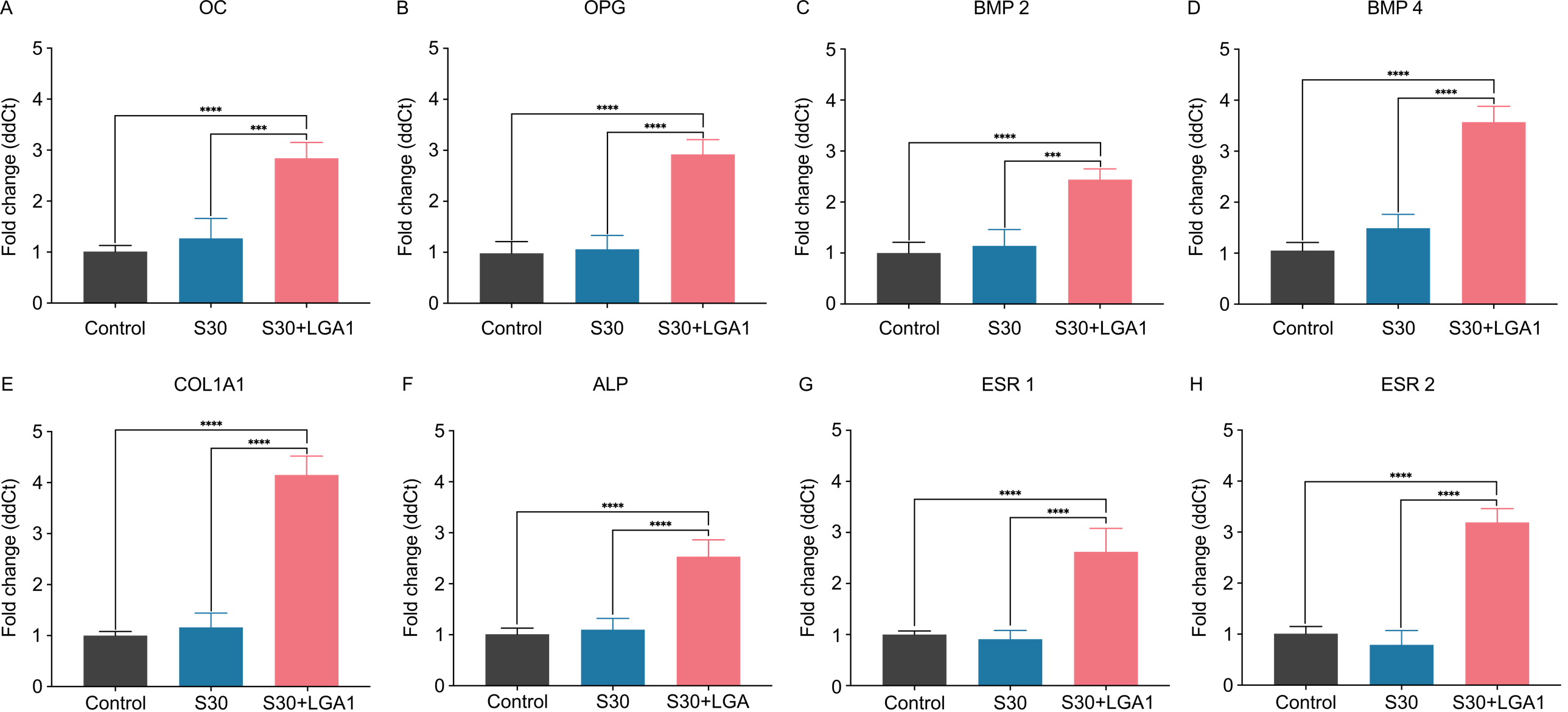


**Figure S3.** Effect of S30+LGA1 on estrogen and bone-related mRNA expression level in MG-63. The gene expression level of (A) OC, (B) OPG, (C) BMP 2, (D) BMP 4, (E) COL1A1 and (F) ALP are used as bone-health makers. The gene expression level of (G) ESR 1 and (H) ESR 2 are used as estrogen markers. After extracting total RNA from the cells, the mRNA levels are quantified using real-time PCR. The obtained values are normalized based on the expression level of β-actin. Data are presented as the mean ± standard deviations (SD). ****p* < 0.001, *****p* < 0.0001 when compared with the control group. S30, soybean germ extract; LGA1, *Lactobacillus gasseri*; OC, osteocalcin; ALP, alkaline phosphatase; OPG, osteoprotegerin; BMP, bone morphogenetic protein; COL1A1, Collagen, type I, alpha 1; ESR, estrogen receptor

**Table S1.** Primer sequences for real-time RT-PCR analysis of mRNA quantification.

| Target gene | Sequences (5' to 3') | |
| --- | --- | --- |
| ESR1 | F | AAT TCA GAT AAT CGA CGC CAG |
|  | R | GTG TTT CAA CAT TCT CCC TCC TC |
| ESR2 | F | TAG TGG TCC ATC GCC AGT TAT |
|  | R | GGG AGC CAA CAC TTC ACC AT |
| pS2 | F | CAT GGA GAA CAA GGT GAT CTG |
|  | R | CAG AAG CGT GTC TGA GGT GTC |
| Osteocalcin | F | ACA CTC CTC GCC CTA TTG |
|  | R | GAT GTG GTC AGC CAA CTC |
| Alkaline phosphatase | F | AAA CCG AGA TAC AAG CAC TCC CAC |
|  | R | TCC GTC ACG TTG TTC CTG TTC AG |
| Osteoprotegerin | F | GGA ACC CCA GAG CGA AAT ACA |
|  | R | CCT GAA GAA TGC CTC CTC ACA |
| Collagen, type I, alpha 1 | F | GCG GCT CCC CAT TTT TAT ACC |
|  | R | GCT CTC CTC CCA TGT TAA ATA GCA |
| Bone morphogenetic protein 2 | F | GCG TGA AAA GAG AGA CTG C |
|  | R | CCA TTG AAA GAG CGT CCA C |
| Bone morphogenetic protein 4 | F | ACG GTG GGA AAC TTT TGA TGT G |
|  | R | CGA GTC TGA TGG AGG TGA GTC |
| β-actin | F | CAT GTA CGT TGC TAT CCA GGC |
|  | R | CTC CTT AAT GTC ACG CAC GAT |

F = forward; R = reverse.
